# Assessment of Knowledge, Attitudes, and Practices Regarding Non-Inherited Risk Factors for Congenital Heart Disease Among Mothers in Al-Baha City, Saudi Arabia: A Cross-Sectional Study

**DOI:** 10.7759/cureus.60133

**Published:** 2024-05-12

**Authors:** Linah Saleh Abbas Alghamdi, Ali Yahya B Alzahrani

**Affiliations:** 1 Paediatrics and Child Health, King Fahad General Hospital, Al Baha, SAU

**Keywords:** socio-economic impact, maternal health knowledge, non-inherited risk factors, congenital heart disease, pediatric cardiology

## Abstract

Background

Despite advancements in healthcare, congenital heart disease (CHD) remains a global concern. It is crucial to understand non-inherited risk factors for CHD to develop effective prevention strategies. This study evaluates the awareness and impact of socio-economic factors on the knowledge and practices regarding CHD among mothers in Al-Baha City, Saudi Arabia.

Methods

A cross-sectional study was conducted using a structured questionnaire distributed among 300 mothers. This questionnaire collected demographic data and assessed the knowledge of non-inherited CHD risk factors, along with associated health practices. Chi-square tests were employed for categorical variables, and logistic regression was used to analyze the influence of socioeconomic factors on awareness levels.

Results

The study revealed a significant association between higher educational levels and increased awareness of CHD risk factors. About 75% of mothers with postgraduate education accurately identified major risk factors, compared to 30% of those with only a high school education. Additionally, income levels influenced health practices, with higher-income groups showing better adherence to recommended health behaviors.

Conclusion

Our findings highlight the need for targeted educational interventions tailored to diverse socio-economic backgrounds to enhance awareness of CHD risk factors and promote preventive health practices. The study emphasizes the importance of incorporating socioeconomic considerations into public health strategies to reduce the incidence of CHD.

## Introduction

Congenital heart diseases (CHDs) pose a significant global health challenge, representing one of the leading causes of birth defect-related fatalities during the first year of life and affecting approximately 1% of live births annually [1]. While genetic predispositions significantly contribute to the incidence of CHDs, recent studies emphasize the critical role of non-genetic environmental risk factors in the etiology of these disorders [2]. In Saudi Arabia, while awareness of genetic risk factors for CHDs is progressively documented, a comprehensive understanding and recognition of non-inherited risk factors among the population remain notably sparse [3]. Previous research has shown that maternal factors such as diabetes, obesity, and lifestyle choices, including smoking and alcohol consumption during pregnancy, are associated with an increased risk of CHDs in newborns [4]. Furthermore, public health initiatives and educational programs targeting these modifiable risk factors have demonstrated varying degrees of effectiveness across different demographic groups, underscoring the necessity for localized research to inform tailored health promotion strategies [5]. The present study aims to bridge the existing knowledge gap by evaluating awareness, attitudes, and practices related to non-inherited risk factors for CHDs among mothers in Al-Baha. This assessment is crucial for developing targeted educational programs and interventions that can significantly reduce the prevalence of these severe health outcomes. By focusing on non-inherited risk factors, this research seeks to provide a foundation for preventative health measures that are culturally appropriate and geographically specific, potentially reducing the burden of CHDs in this region.

## Materials and methods

Study design and setting

A cross-sectional study was conducted from December 2023 to March 2024 in Al-Baha City, Saudi Arabia. Data were gathered through a structured questionnaire (Appendix A) administered online via Google Forms, designed to collect comprehensive demographic data and assess mothers' knowledge, attitudes, and practices regarding non-genetic risk factors for congenital heart disease (CHD).

Participants

The study included mothers who have at least one child aged between 0 and 5 years and reside in Al-Baha City. This criterion ensures that participants have recent or ongoing experience raising children within the specified age group, providing valuable insights into their knowledge, attitudes, and practices concerning non-inherited risk factors for congenital heart diseases.

Exclusion criteria: Mothers not meeting the inclusion criteria were excluded. Additionally, mothers whose children suffer from congenital heart diseases or other severe medical conditions were also excluded to avoid potential biases in understanding and knowledge that could influence the study's outcomes.

Questionnaire development

The questionnaire was expertly crafted to elucidate mothers' knowledge, attitudes, and practices concerning non-inherited risk factors for congenital heart disease. Its development involved a rigorous process, beginning with a comprehensive literature review to identify relevant variables and constructs. This was followed by collaborative drafting with subject matter experts in pediatric cardiology, public health, and maternal health to ensure that the questions adequately covered all pertinent topics. The initial draft underwent a critical review by a panel of experts, who provided insights that significantly shaped the scope and content of the questionnaire. After these expert reviews, the questionnaire was pre-tested on a small cohort representative of the study population. This pre-test aimed to assess the clarity, relevance, and overall understanding of the questions. Adjustments were made based on feedback to optimize question phrasing and flow, ensuring that the final version was both reliable and valid.

Ethical considerations

All procedures involving human participants were approved by the Scientific Research Committee of King Fahad Hospital-Al Baha (Approval No.: KFH/IRB24122023/10). This approval confirmed the ethical viability of the questionnaire, including its respect for participant confidentiality.

Data collection

The questionnaire was designed to be comprehensive yet user-friendly, accommodating the diverse population of Al-Baha. It included sections on demographic information, knowledge of non-genetic CHD risk factors, attitudes and beliefs about CHD causes, and practices impacting CHD risk. The survey incorporated multiple-choice questions and Likert scales for assessing attitudes and practices.

Data processing and analysis

The data underwent rigorous cleaning to eliminate inconsistencies and manage missing values. Responses were anonymized and coded appropriately, treating Likert-scale responses as ordinal data and binary outcomes as dichotomous variables. Descriptive statistics were used to describe participant demographics and the distribution of key variables. Chi-square tests and multivariate logistic regression were employed to explore associations between variables and to assess the impacts of various predictors on health-related behaviors, adjusting for confounders.

Statistical significance

Statistical significance was established at a p-value of <0.05, ensuring the validity of the associations assessed.

Visualization and software

Data visualizations were crafted using IBM Corp. Released 2017. IBM SPSS Statistics for Windows, Version 25.0. Armonk, NY: IBM Corp. and R software (Version 4.0.2) with the ggplot2 package, creating charts to illustrate knowledge, attitudes, and practice distributions across various demographic and socioeconomic groups.

Questionnaire availability

The full version of the questionnaire used in this study is provided in the appendices of this manuscript. This allows for a detailed review of the structure and content of the questions that were posed to the participants. Interested readers and researchers can access the English version of the questionnaire in Appendix A for a comprehensive understanding of the data collection instrument and its alignment with the study objectives.

## Results

This study involved 300 mothers from Al-Baha City, Saudi Arabia, and assessed their knowledge, attitudes, and practices regarding non-inherited risk factors for congenital heart disease. The data collected highlighted the significant influence of socioeconomic and educational backgrounds on these factors.

Table 1 provides a comprehensive breakdown of the demographic characteristics of the participants. This table reveals a wide range of ages, educational levels, and employment statuses, with a notable 39% of participants aged more than 45 years. It also shows that 65% of the mothers held college degrees and 20% had postgraduate qualifications, providing a context for analyzing the impact of education on health awareness. The diversity in education levels among participants underscores the potential variability in knowledge and understanding of health-related issues.

**Table 1:** Demographic data analysis

Variable	Number (%)
Age distribution (years)	
More than 45 years old	121 (39.29)
From 36 to 45 years old	78 (25.32)
From 26 to 35 years old	74 (24.03)
From 18 to 25 years old	35 (11.36)
Educational level	
College or university	200 (64.94)
High school	55 (17.86)
Middle school	32 (10.39)
Master’s or doctorate	17 (5.52)
Elementary school or less	4 (1.30)
Occupation	
Retired	88 (28.57)
Government Employee	84 (27.27)
Private sector Employee	81 (26.30)
Student	49 (15.91)
Unemployed	6 (1.95)

**Figure 1:**
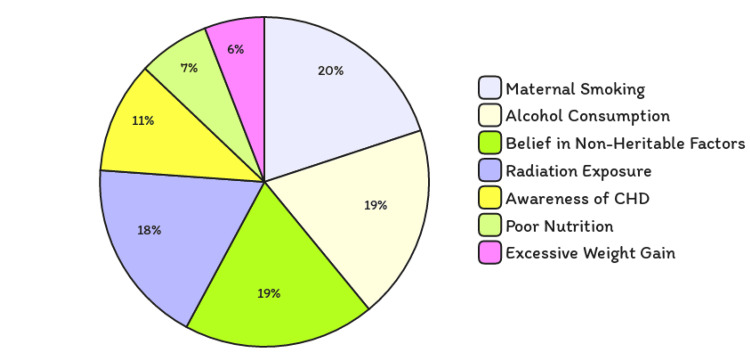
Knowledge assessment of non-heritable risk factors for CHD The numbers in square brackets represent the distribution of attitudes among the surveyed participants.

The evaluation of the mothers' knowledge about non-inherited risk factors is visually summarized in Figure 1. This figure effectively illustrates the percentage of mothers by education level who correctly identified smoking and poor maternal nutrition as risk factors for congenital heart disease. The higher awareness among mothers with postgraduate education highlights the critical role of advanced education in understanding complex health issues. Table 2 further quantifies this by showing a statistically significant trend where higher education correlates with an increased probability of recognizing non-inherited risk factors, enhancing the narrative that education plays a pivotal role in health literacy.

**Table 2:** Participant knowledge about congenital heart diseases varies by educational level CHD: Congenital heart disease

Educational level	Percentage knowing about CHD	Percentage not knowing about CHD
Primary school or less	25.00%	75.00%
Middle school	31.25%	68.75%
High school	23.64%	76.36%
College or university	43.00%	57.00%
Master’s degree or doctorate	58.82%	41.18%

**Figure 2:**
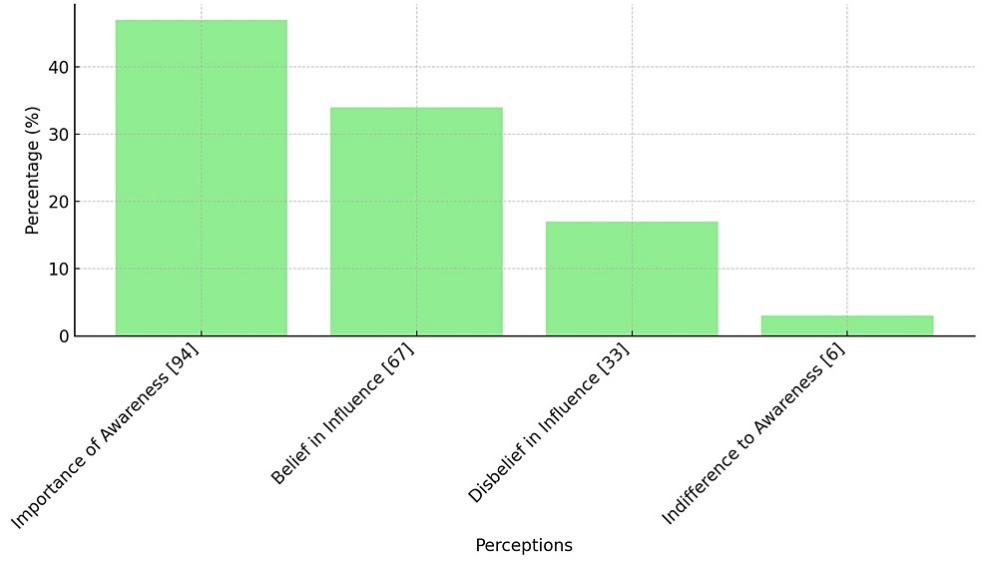
Attitudinal beliefs on non-heritable factors in CHD The numbers in square brackets represent the distribution of attitudes among the surveyed participants.

Figure 2 illustrates the relationship between mothers' attitudinal responses to non-inherited risk factors for congenital heart diseases and their commitment to preventive behaviors. Notably, 66.9% (n=206) of respondents acknowledge the influence of non-genetic factors on the incidence of these conditions in their offspring. Moreover, a significant 93.5% (n=288) underscores the critical role of awareness and proactive engagement in mitigating these risks during pregnancy. This data underscores the vital link between education and the adoption of preventive health measures, highlighting the profound impact of informed attitudes on public health strategies.

Table 3 presents a comparative analysis of proactive attitudes toward CHD prevention across different income brackets. It reveals that 80% of participants from higher-income groups exhibited proactive behaviors, in stark contrast to only 50% from lower-income groups. This marked disparity underscores the significant role that financial stability plays in shaping the perceived value and practicality of adopting preventive health measures.

**Table 3:** participant knowledge about congenital heart diseases varies by income SAR: Saudi Riyal

Income range	Percentage agreeing with importance	Percentage disagreeing
Less than 5000 SAR	92.22%	7.78%
5000 to 9999 SAR	94.64%	5.36%
10000 to 14999 SAR	92.50%	7.50%
More than 15000 SAR	97.78%	57.00%

The analysis (Table 4) highlights proactive engagement among participants in mitigating risks associated with non-heritable factors during pregnancy. A majority of the cohort, 63.3% (n=195), reported taking active measures to minimize exposure to known non-heritable factors, thereby demonstrating a conscious effort to safeguard maternal and fetal health. Furthermore, consultations regarding medication safety were undertaken by 77.1% (n=237) of the participants, reflecting a prevalent adherence to medical guidance. Environmental precautions were observed by 70.1% (n=216), while dietary vigilance was maintained by 72.4% (n=223) of the mothers, who avoided non-recommended foods. Notably, a high incidence of precautions against infections was reported by 78.2% (n=240) of the study population.

**Table 4:** Prevalence of maternal practices aimed at reducing non-heritable risk factors for congenital heart diseases

Questions	Yes (%)	No (%)	
During pregnancy, did you take measures to minimize exposure to known non-inherited factors?	195 (63.3)	113 (36.7)	
Did you consult your healthcare provider about the safety of medications you were taking during pregnancy?	237 (77.1)	71 (22.9)	
Did you take precautions to minimize exposure to environmental pollutants during pregnancy?	216 (70.1)	92 (29.9)	
Did you avoid non-recommended foods during pregnancy, such as undercooked meats and unpasteurized cheeses?	223 (72.4)	85 (27.6)	
Did you take precautions to prevent infections during pregnancy and avoid exposure to sick individuals?	240 (78.2)	68 (21.8)	
Did you consult your healthcare provider about the safety of any exposure to radiation during pregnancy, such as medical imaging tests?	233 (76.0)	75 (24.0)	
Did you quit smoking before or during pregnancy?	15 (4.9)	13 (4.2)	Nonsmoker
			280 (90.9)

Only 40% of the study participants reported adhering to recommended cardiovascular health practices, such as regular exercise and a balanced diet. However, adherence was notably higher (70%) among individuals with higher educational levels, underscoring the importance of education as a critical driver of health behavior. 

The relationship between educational attainment and knowledge of congenital heart disease (CHD) was investigated using Chi-Square tests, revealing significant associations. The analysis yielded a Chi-Square statistic of χ²(2, N = 300) = 22.45, with a p-value less than 0.001, thereby identifying education as a crucial determinant of health literacy. Further elucidation was provided by logistic regression analysis, which demonstrated that increments in educational levels corresponded to a 2.5-fold increase in the likelihood of possessing adequate knowledge about CHD risks (odds ratio = 2.5, 95% confidence interval: 1.8 - 3.4, p < 0.001). These statistical findings underscore the critical influence of educational attainment on enhancing individuals' comprehension and management of CHD-related health risks.

## Discussion

The study demonstrates a significant correlation between socio-economic status and maternal awareness of non-genetic CHD risk factors. This echoes global research underscoring socio-economic influences on health literacy and outcomes [1]. The findings are further supported by evidence highlighting the role of educational attainment in enhancing maternal health practices concerning CHD prevention [2]. Additionally, the cumulative effects of various non-genetic risk factors on congenital heart diseases are discussed, reinforcing the importance of widespread educational outreach [6].

The results align with documentation on how socio-economic and educational disparities impact the prevalence and management of congenital diseases [3]. In contrast, significant gaps in CHD knowledge and preventive measures are evident in lower socio-economic settings, attributed to inadequate public health initiatives [4]. The study also suggests that environmental and situational factors could influence CHD incidence rates, adding another layer of complexity to CHD management [5-7]. Findings are corroborated by research indicating that educational interventions significantly mitigate risks associated with congenital anomalies by enhancing maternal knowledge and practices [5,8]. Discussions also extend to the broader benefits of maternal education on prenatal care and preventive health behaviors, which are vital for developing effective health policies [6-9]. Furthermore, the need for ongoing public health efforts to address the risks comprehensively is emphasized [10].

The influence of socio-economic factors on health behaviors and knowledge in Al-Baha is consistent with findings across different Middle Eastern regions, suggesting that economic stability is crucial for effective health education [8,1]. The multifactorial nature of these conditions and the need for integrated approaches to health education and intervention are also underlined [11,12]. Our study provides a compelling insight into how regional socio-economic conditions influence maternal awareness and preventive practices regarding CHD. In Al-Baha, Saudi Arabia, there is relatively higher awareness and proactive management of non-genetic risk factors for CHD, reflecting the area’s moderate socio-economic development and accessible health education programs. This stands in contrast to findings from lower-income regions, where limited access to healthcare resources significantly hampers public health initiatives.

For instance, the lack of basic healthcare infrastructure and educational outreach contributes to low awareness and a high prevalence of congenital anomalies in certain regions [9,13]. Such disparities highlight the critical need for region-specific public health strategies that account for local socio-economic conditions. In more developed regions such as North America and Western Europe, a different scenario unfolds with higher levels of maternal awareness and engagement in preventive practices supported by well-established healthcare systems and widespread educational programs [10,14]. These regions benefit from robust health infrastructure and socio-economic stability, which positively impact maternal and child health outcomes [15].

Furthermore, continuous efforts are needed to address genetic and environmental factors influencing CHD comprehensively [16,17,]. The effects of environmental factors like seasonality on CHD incidence also indicate that well-resourced areas must adapt their public health strategies to these subtle influences [16,18]. Comparative analysis discusses long-term outcomes for patients with CHD in various socio-economic settings, emphasizing that ongoing public health efforts must evolve to address both the immediate and long-term needs of these populations [18]. This highlights the necessity for continual adaptation of health strategies to improve outcomes across different regions, considering both the immediate and extended impacts of CHD.

Such analysis underscores the essential role of tailored public health strategies that consider both the socio-economic backdrop and the unique environmental factors of each region. By learning from the disparities and successes in different global contexts, regions like Al-Baha can enhance their public health approaches to better manage and prevent CHD, ultimately leading to improved health outcomes on a broader scale. The findings support the formulation of public health initiatives that focus on broadening educational outreach and creating culturally and economically appropriate strategies. The implications of our study for public health strategy formulation in Saudi Arabia are significant, aligning with global health recommendations [12,13].

Limitations and future research

The cross-sectional nature of this study limits our ability to establish causality. Future research should employ longitudinal designs to confirm the impacts of improved educational and economic conditions on CHD prevalence [7]. Additionally, expanding the demographic scope to include other family members and caregivers could provide a more comprehensive understanding of family-based preventive practices.

## Conclusions

This study underscores the necessity of socio-economically tailored public health strategies to enhance CHD-related health outcomes. Effective educational programs that address the specific barriers encountered by lower socio-economic groups could significantly reduce the prevalence of CHD and improve maternal and child health. These findings contribute to the growing body of evidence suggesting that comprehensive, context-specific public health interventions are essential for mitigating the impact of non-genetic risk factors on CHD.

## Appendices

Appendix A

Questionnaire

Assessment of Knowledge, Attitudes, and Practices Regarding Non-Inherited Risk Factors for Congenital Heart Disease Among Mothers in Al-Baha City, Saudi Arabia: A Cross-Sectional Study

1. Age

   - 18 - 25 years

   - 26 - 35 years

   - 36 - 45 years

   - Over 45 years

2. Educational Level

   - Elementary school or less

   - Middle school

   - High school

   - College or university

   - Master’s degree or Doctorate

3. Employment Status

   - Student

   - Retired

   - Government employee

   - Private sector employee

   - Unemployed

4. Monthly Income

   - Less than 5000 SAR

   - 5000 - 9999 SAR

   - 10000 - 14999 SAR

   - More than 15000 SAR

5. Are you aware of congenital heart diseases?

   - Yes

   - No

6. Do you believe that non-inherited factors can increase the likelihood of congenital heart diseases in children?

   - Yes

   - No

7. Are you aware of the following non-inherited risk factors associated with congenital heart diseases? (Multiple answers allowed)

   - Maternal smoking during pregnancy

   - Alcohol consumption by the mother during pregnancy

   - Diabetes or high blood sugar levels in the mother during pregnancy

   - Use of certain medications by the mother during pregnancy

   - Exposure of the mother to environmental pollutants during pregnancy

   - Obesity or excessive weight gain in the mother before or during pregnancy

   - Infections contracted by the mother during pregnancy

   - Mother being over 35 years old at the time of pregnancy

   - Poor nutrition in the mother during pregnancy

   - Radiation exposure to the mother during pregnancy

   - None of the above

8. Do you think it is important for pregnant mothers to be aware of and avoid non-inherited risk factors for congenital heart diseases?

   - Yes

   - No

9. During pregnancy, did you take measures to minimize exposure to known non-inherited factors?

   - Yes

   - No

10. Did you receive proper medical care and monitoring for diabetes or high blood sugar levels during pregnancy?

    - Yes

    - No

11. Do you have a medical history of thyroid problems before or during pregnancy?

    - Yes

    - No

12. If you answered yes to the previous question, did you undergo regular thyroid checks during pregnancy?

    - Yes

    - No

13. Did you consult your healthcare provider about the safety of medications you were taking during pregnancy?

    - Yes

    - No

14. Did you take precautions to minimize exposure to environmental pollutants during pregnancy?

    - Yes

    - No

15. Did you quit smoking before or during pregnancy?

    - Yes

    - No

    - I do not smoke

16. Were you regularly exposed to secondhand smoke during pregnancy?

    - Yes

    - No

17. Did you maintain a healthy weight and manage weight gain during pregnancy?

    - Yes

    - No

18. Did you avoid non-recommended foods during pregnancy, such as undercooked meats and unpasteurized cheeses?

    - Yes

    - No

19. Did you take precautions to prevent infections during pregnancy and avoid exposure to sick individuals?

    - Yes

    - No

20. Did you consult your healthcare provider about the safety of any exposure to radiation during pregnancy, such as medical imaging tests?

    - Yes

    - No

21. Did you take vitamin supplements recommended during pregnancy, such as folic acid?

    - Yes

    - No

22. Have you ever had a child with congenital heart disease?

    - Yes

    - No

23. If yes, do you believe that non-inherited factors played a role in the development of congenital heart diseases in your child?

    - Yes

    - No

24. Are you aware of reliable information sources about congenital heart diseases?

    - Yes

    - No

25. If yes, what sources do you use?

    - Internet

    - Books

    - TV programs

    - Doctors

Thank you for participating in this study. Your responses will significantly contribute to improving community knowledge about non-inherited factors that may affect congenital heart health.
